# Simulation in pediatric minimally invasive surgery: adaptation of a simple series of exercises as part of the process of an initial implementation of a resident training program

**DOI:** 10.1590/0100-6991e-20243574-en

**Published:** 2024-05-02

**Authors:** ELISÂNGELA DE MATTOS E SILVA, ARNON CÉSAR BRUNET SCHULTZE, BRUNO BERARDI GAZOLA, AMANDA GINANI ANTUNES, KARIN LUCILDA SCHULTZ, FERNANDO ANTÔNIO BERSANI AMADO

**Affiliations:** 1 - Hospital Pequeno Príncipe, Departamento de Cirurgia Pediátrica - Curitiba - PR - Brasil; 2 - Faculdades Pequeno Príncipe, Curso de Medicina - Curitiba - PR - Brasil

**Keywords:** Minimally Invasive Surgical Procedures, Simulation Exercise, Laparoscopy, Simulation Training, Cirurgia Vídeo Assistida, Exercício de Simulação, Laparoscopia, Treinamento por Simulação, Pediatria

## Abstract

**Introduction:**

the simulation in minimally invasive surgery is fundamental for surgeon in training to learning and training skills, especially in pediatrics, due to the particularities, reduced spaces, specific and rare procedures. The aim of this study was to propose an adapted series of exercises and to simply evaluate the performance of pediatric surgery residents in the initial implementation of a training program.

**Method:**

seven basic skills exercises in video surgery, based on series and programs already published and using low-cost materials, were performed by six residents in 2 moments, with an interval of 15 days and evaluated by simple instrument.

**Results:**

there was no difficulty with models. Considering the individual averages of the seven exercises together in the two moments, five of the six residents increased the score in the second moment. The average score per exercise increased in five of the seven tasks. Despite the small number of participants and repetition, it has already been possible to observe a trend of better performance with decreased time of all residents after a single repetition. All considered the exercises capable of training essential skills of the specialty, with simple and inexpensive materials.

**Conclusion:**

given the challenges of simulated training in pediatric video surgery, it is known the benefit of a continuous program, with exercises that can simulate real situations. A pre-established schedule, more participants and repetitions, supervision of experienced surgeons and validated instruments are fundamental to evaluate surgeons in training and show statistical benefits of simulated exercises in this series.

## RESUMO

**Introdução:**

a simulação em cirurgia minimamente invasiva é fundamental para treinamento e aprendizagem de habilidades ao cirurgião em formação, especialmente na pediatria, devido às particularidades da especialidade, espaços reduzidos, procedimentos específicos e raros. O objetivo deste estudo foi propor uma adaptação de uma série de exercícios em simulador e avaliar o desempenho dos residentes de cirurgia pediátrica na implementação inicial de um programa de treinamento.

**Método:**

sete exercícios de habilidades básicas em videocirurgia, baseados em séries e programas já publicados e utilizando materiais de baixo custo, foram realizados por seis residentes em 2 momentos, com intervalo de 15 dias e avaliados por instrumento simples.

**Resultados:**

não houve dificuldade em relação aos modelos. Considerando- se as médias individuais dos sete exercícios juntos nos dois momentos, cinco dos seis residentes aumentaram a pontuação no segundo momento. A pontuação média por exercício aumentou em cinco das sete tarefas. Apesar do pequeno número de participantes e repetição, já foi possível observar uma tendência de melhor desempenho com diminuição do tempo de todos os residentes após uma única repetição. Todos consideraram os exercícios capazes de treinar habilidades essenciais da especialidade, de maneira acessível e com materiais simples e baratos.

**Conclusão:**

diante dos desafios do treinamento simulado em videocirurgia pediátrica, sabe-se do benefício de um programa contínuo, com exercícios que simulam situações reais. Um cronograma pré-estabelecido, mais participantes e mais repetições, supervisão de cirurgiões experientes e instrumentos validados são fundamentais para avaliar a evolução dos cirurgiões em formação e permitir demonstrar resultados com significância estatística.

## INTRODUCTION

Simulation has been increasingly used as a strategy to allow the learning and training of skills and competencies necessary for professional performance, in a controlled and safe environment for the surgeon and the patient. The possibility of carrying out procedures repeatedly on mannequins and simulators allows errors to be corrected, in addition to enabling technical evolution to be monitored^1^. Aiming to improve skills and minimize risks, simulation training has been highly valued in the health sector, at different levels of training, mainly in the surgical area involving minimally invasive surgeries^2^.

Especially in pediatric surgery, training must include learning complex and rare procedures, neonatal malformations, oncological surgeries, as well as video surgical skills in small cavities, handling delicate tissues and improving finer and more precise movements. Therefore, safe training is even more necessary due to the particularities of the specialty, which has specific procedures and fewer cases in daily practice.

Several simulation models in pediatric minimally invasive surgery have been described in the world literature, reinforcing the advantages of safe training in an appropriate environment, with the possibility of repetitions and without the limitations and risks of in vivo training. However, in Brazil most pediatric surgery residents unfortunately still develop their skills on the job, as they assist and perform procedures under supervision, directly on patients^3^. Simulated basic skills training models in video surgery, using inanimate (black box) or virtual simulators, allow for the improvement of depth and distance perception, bimanual dexterity, optical and tissue manipulation, and knowledge of materials. The most advanced models allow the reproduction of specialty-specific diseases as close as possible to reality^4^.

Considering the scarcity of official training programs focused on the area of minimally invasive surgery in Brazil^5^, mainly in pediatric surgery, the objective of this work is to propose an adaptation of a series of basic black box exercises and evaluate the performance of residents of a pediatric surgery service in simulated video surgery training as an initial phase of the process of implementing a training program in this service.

## METHODS

We developed and applied a series of seven exercises to all six residents, two from each year of residency, at the Pediatric Surgery Service of Hospital Pequeno Príncipe, Curitiba, Paraná, Brazil. The series of exercises was based on and inspired by other series and by the North American Fundamentals of Laparoscopic Surgery (FLS) program described in the literature, adapting some exercises to the availability and viability of the models. Another series of exercises, created, self-applied and self-evaluated by the residents themselves years before, also served as inspiration and basis for the current series^6^.

The six residents received instructions and performed the same series of exercises in two moments (“series 1” and “series 2”), with an interval of approximately 15 days, using a video surgery simulator with a camera (black box), a basic set of laparoscopic instruments (including Maryland, scissors, needle holder, and grasping forceps), one monitor - 21.5” screen, portable capture plate (Recorder), surgical sutures (nylon 4.0 and polygalactin 3.0), and acrylic and polystyrene bases, with the models developed by the researchers themselves.

**Figure 1:**
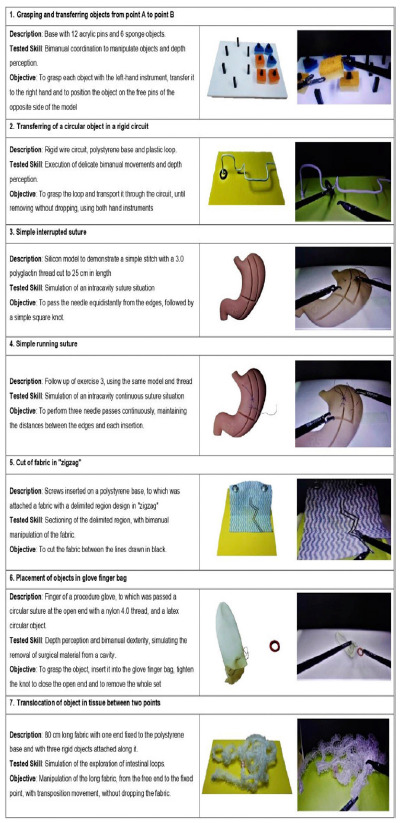
Description and illustration of the exercises.

The seven exercises performed are described and illustrated with original photos in Figure 1. The models used are easy to reproduce with simple materials and can be adapted and reproduced in any service. Even exercises 3 and 4 that used a model with a specific shape can be replaced by any structure that allows simple or continuous suturing between two edges, such as models latex balloons.

The skills assessed were grasping and transferring objects, transferring a circular object (ring) in a rigid circuit, simple interrupted suture, simple continuous suture, cutting tissue in a pre-defined area, placing objects in a glove finger bag, and translocation of a piece of tissue between two points.

**Figure 2:**
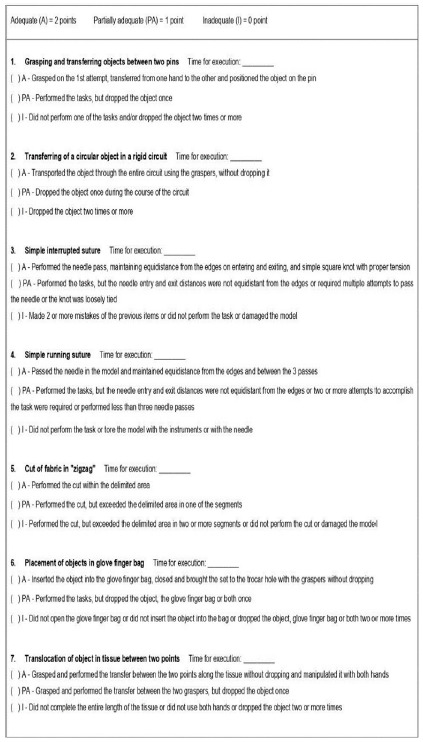
Exercise evaluation instrument.

An evaluation instrument containing the objectives of the exercises and assigning grades to each one of them was developed by the authors and filled by the researchers to evaluate the residents’ performance in these two moments of activity, as illustrated in Figure 2. The instrument used was prepared by the authors and adapted for the chosen exercises, as a way of serving as a reference and collecting some objective data regarding the residents’ performance.

Data were collected and tabulated in Microsoft Excel^®^ spreadsheets and analyzed using the Statistical Package for the Social Science - SPSS^®^ computer software (IBM^®^ SPSS^®^ Statistics v. 25.0, SPSS Inc, Chicago, USA). The results were expressed as means (quantitative variables). For inferential analyzes, we used the Wilcoxon Non-Parametric Test, considering values of p<0.05 as significant.

This study was conducted after approval by the institution’s Ethics and Research Committee, under registration CAAE 03943218.9.0000.5580, and followed the ethical recommendations for research in Ordinance 466/12 of the National Health Council. All participants signed an informed consent form.

## RESULTS

All six pediatric surgery residents at Hospital Pequeno Príncipe at Curitiba participated in the study. There were two residents from each year of specialization, and all performed the series of exercises at the two proposed times and with an interval of approximately 15 days between them, varying from 14 to 17 days, according to guidance.

There were no difficulties or problems related to the proposed models and exercises, all of which were easy for participants to understand and evaluate during the study.

Table 1 shows the complete results spreadsheet with the time and scores of the six residents in the seven exercises performed.

**Table 1:** Results spreadsheet.

			Resident 1	Resident 2	Resident 3	Resident 4	Resident 5	Resident 6
EXERCISE 1	Series 1	Score	2	2	2	1	2	0
		Time	0:02:01	0:01:40	0:01:59	0:02:19	0:01:37	0:02:42
	Series 2	Score	1	2	1	2	2	2
		Time	0:02:59	0:01:47	0:03:18	0:01:50	0:01:46	0:01:54
EXERCISE 2	Series 1	Score	0	0	0	1	0	1
		Time	0:01:26	0:01:53	0:01:41	0:02:22	0:03:29	0:00:49
	Series 2	Score	0	1	1	1	0	2
		Time	0:02:25	0:01:43	0:01:58	0:01:03	0:02:51	0:00:37
EXERCISE 3	Series 1	Score	1	1	1	2	1	2
		Time	0:20:33	0:09:05	0:07:12	0:04:36	0:07:55	0:03:38
	Series 2	Score	2	1	2	2	1	2
		Time	0:05:31	0:05:30	0:05:00	0:03:14	0:04:20	0:03:08
EXERCISE 4	Series 1	Score	1	1	1	1	1	2
		Time	0:08:29	0:02:24	0:05:00	0:04:08	0:02:18	0:02:46
	Series 2	Score	0	1	2	1	1	1
		Time	0:07:33	0:03:03	0:01:44	0:02:20	0:01:56	0:03:20
EXERCISE 5	Series 1	Score	2	2	1	2	0	^1^
		Time	0:03:51	0:02:17	0:04:30	0:04:08	0:01:49	0:02:15
	Series 2	Score	0	2	2	2	1	2
		Time	0:08:26	0:02:30	0:03:45	0:02:38	0:02:17	0:02:31
EXERCISE 6	Series 1	Score	2	2	2	2	2	2
	Time	0:00:51	0:00:36	0:00:56	0:00:54	0:00:49	0:00:43
	Series 2	Score	2	2	2	2	2	2
		Time	0:00:54	0:00:36	0:00:49	0:00:40	0:00:51	0:00:23
EXERCISE 7	Series 1	Score	1	2	2	2	1	2
		Time	0:07:21	0:03:24	0:05:52	0:03:07	0:03:41	0:03:53
	Series 2	Score	2	2	2	2	2	2
		Time	0:04:05	0:03:24	0:04:22	0:05:18	0:04:03	0:03:05

**Figure 3:**
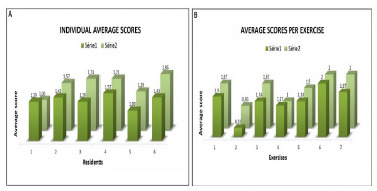
Average grades in both series. Graph A. Average per resident in all exercises. Graph B. Average in each exercise for all residents.

Considering the scores, when comparing the participants’ individual averages in the seven exercises in both series, five of the six residents increased their scores from the first to the second time they performed the exercises, as illustrated in graph A (Figure 3).

As for the average scores of all residents per exercise in the two moments, when comparing the second moment with the first, a higher result was obtained in five exercises, lower in one exercise (exercise 4) and stayed the same in one (exercise 6). (Graph B - Figure 3)

All these values were not statistically significant, with p-values>0.05. Only exercise 2 showed a tendency towards significance between the two repetition moments of the series (p=0.083).

The exercise with the lowest average was number 2 (transferring a circular object in a rigid circuit), with an average score of 0.33 the first time and 0.83 the second time. The exercise with the highest average score was number 6 (placing an object in a glove finger bag), with an average of 2.0 (maximum score) in both series.

**Figure 4:**
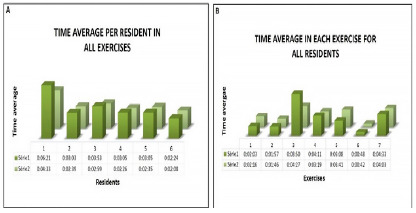
Average times in both series. Graph A. Average per resident in all exercises. Graph B. Average in each exercise for all residents.

All residents completed series 2 in less time than series 1 (Graph A - Figure 4). Graph B in Figure 4 shows the average execution times for each resident in the two series. The first series was performed in an average total time of 25 minutes and 30 seconds per resident. In the second, this time decreased to 20 minutes and 15 seconds, a reduction of approximately 20%. The fastest exercise was number 6 (average of 48 seconds in the first series and 42 seconds in the second) and the one that took longer was number 3 (average of 8 minutes and 50 seconds in the first series and 4 minutes and 27 seconds in second). Exercise 3 was also the only one with statistical significance when we carried out a comparative analysis of time between the first and second series (p=0.027), with a proven reduction in time.

**Figure 5:**
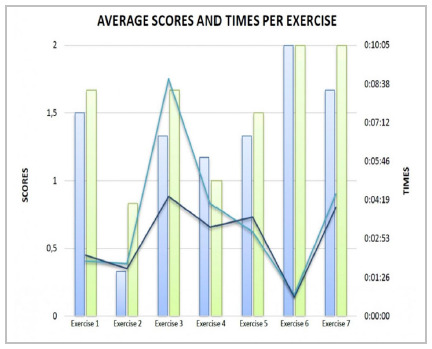
Average scores represented in lines (series 1 dark blue and series 2 light blue). Average times represented in columns (blue series 1 and green series 2) per exercise.

Considering the residents’ performance with the execution times in both series, exercise 6 was performed faster and with higher scores in both the first and second moments. Exercise 3, despite having the longest average execution time, presented relatively high average scores (average of 1.33 in the first series and 1.67 in the second). Exercise 2, despite having the lowest average score, was the second fastest exercise to be performed (average of 1 minute and 57 seconds in the first series and 1 minute and 46 seconds in the second) (Figure 5).

Due to the small number of participants and repetition of the series, it was not possible to confirm better performance and time results with statistical analysis in the second series in relation to the first, although this trend was clear in the results presented in graphs and numbers even with few repetitions and reduced sample.

## DISCUSSION

Surgical training in a simulated environment improves movements, trains skills, and reduces the risk of complications^7^. Minimally invasive surgery requires more time to learn basic skills^8^ compared with open surgery, also requiring more training time.

Among the basic specific skills required to perform video surgery procedures, there are visual adaptation, notion of depth in a two-dimensional image, and motor skills to manipulate instruments and structures appropriately.

Skill acquisition is divided into three stages: 1) Initial (cognitive), with inconsistent movements that require a high degree of attention; 2) Intermediate (associative), with few serious errors, requiring a lower level of attention; 3) Advanced (autonomous), in which there is greater agility, allowing the execution of simultaneous tasks, reaching a level of proficiency^4^.

Several training programs with similar structures and different exercises have been created and others are constantly being developed to train and improve the skills needed for minimally invasive surgery. The Laparoscopic Surgical Skills Program (LSS) is an European training program that evaluates simulation based on performance indicators in the surgical field, considering tests of cognitive, technical, and judgment skills^8^. The Fundamentals in Laparoscopic Surgery (FLS), approved by the American College of Surgeons^9^, have become mandatory for first-year surgery residents, including theoretical content and supervised hands-on exams, allowing surgeons in training to evaluate and record their own skills^10^ ^,^ ^19^ ^-^ ^21^.

In Brazil, pediatric surgeons undergo a period of training in general surgery as a prerequisite for residency in pediatric surgery, but do not always receive specific training in minimally invasive surgery. Even if they have received training, the pediatric surgery resident faces challenges specific to the specialty, such as different pathologies, congenital malformations, and surgeries in small cavities, which require new skills and constant training.

The exercises in this study were created based on existing programs and adaptations of the exercises proposed by the FLS program, and the proposed series proved to be feasible and reproducible, with cheap, simple, and easy-to-construct models, and can be considered another available option for teaching and training during the formation of laparoscopy surgeons. These exercises were created with low-cost materials, showing that training basic skills is possible even without sophisticated and expensive materials.

Some articles indicate a minimum number of repetitions of an exercise or activity to achieve certain skills, such as at least 30 repetitions for each task^7^ or around five sessions in a virtual reality simulator to reach a relatively stable level of knowledge^11^ ^,^ ^12^.

In the present study, in just two sessions with an interval of two weeks, it was possible to observe a trend towards better performance and a reduction in the time spent performing the exercises with repeated practice. We emphasize that the reduced sample does not allow for the possibility of conclusions based on statistics. We also reinforce that the series was inspired and adapted from other already validated series, with the aim of publicizing the possibility of adaptations to enable the training of laparoscopic skills and to encourage the initial implementation of training programs.

The possibility of replicating, in an adapted way, simulation exercises for video surgery skills training, allowed residents to practice and self-evaluate their performance, as well as resulting in better performance observed in some exercises after the second repetition. Although we could not statistically confirm this due to the sample size, it attests to the importance of continued training in a safe and protected environment.

## CONCLUSIONS

Faced with the challenges of promoting effective learning of skills in pediatric minimally invasive surgery, we highlight the benefit of a continuous training program in laparoscopic surgery for residents, with a series of exercises that can be easily reproduced and repeated in simulating real situations, such as those developed in this study. A series of simple exercises with low-cost materials can be easily created for basic skills training.

A pre-established schedule with specific objectives, supervision by trained surgeons, and officially validated assessment instruments are essential to analyze the learning curve in each skill taught and ensure training effectiveness.
